# Subjective attitudes moderate the social connectedness in esports gaming during COVID-19 pandemic: A cross-sectional study

**DOI:** 10.3389/fpubh.2022.1020114

**Published:** 2023-01-04

**Authors:** Dan Shan, Jilai Xu, Tongyu Liu, Yanyi Zhang, Ziyun Dai, Yuandian Zheng, Chang Liu, Yuanning Wei, Zhihao Dai

**Affiliations:** ^1^Department of Biobehavioral Sciences, Teachers College, Columbia University, New York, NY, United States; ^2^School of Medicine, Juntendo University, Tokyo, Japan; ^3^LGD Gaming, Hangzhou, China; ^4^Faculty of Medicine and Health, The University of Sydney, Camperdown, NSW, Australia; ^5^Minhang Crosspoint Academy at Shanghai Wenqi Middle School, Shanghai, China; ^6^College of Osteopathic Medicine, Kansas City University, Kansas City, MO, United States; ^7^School of Clinical Medicine, University of Cambridge, Addenbrooke's Hospital, Cambridge, United Kingdom; ^8^College of Engineering, Cornell University, Ithaca, NY, United States; ^9^School of Medicine, Royal College of Surgeons Ireland, University of Medicine and Health Sciences, Dublin, Ireland

**Keywords:** attitude, communication frequency, COVID-19, online game, social connection, public mental health

## Abstract

**Background:**

Electronic sports (esports) has become a practical intervention for young people craving social connections since the COVID-19 pandemic. Past studies have shown an equivocal role of esports participation in boosting social ties or social connectedness. It is unclear if their relationship is affected by subjective attitudes of gamers. Moreover, the present COVID-19 pandemic may further modify this relationship to a greater extent.

**Objective:**

This study primarily aimed to investigate the moderating effect of participants' subjective attitudes toward esports gaming on the relationship between in-game interaction during esports participation and participants' anticipated social connectedness among Chinese young adults during the COVID-19 lockdown periods in China.

**Methods:**

We conducted a nationwide online questionnaire survey through the Credamo platform among 550 Chinese young adults in the present study. The Social Connectedness Scale-Revised was used to assess participants' social connectedness levels.

**Results:**

Four hundred and fifty-three participants were included in the final analysis. The effective response rate was 82.4%. Our results showed that the esports participation measured by in-game communication frequency among participants, as an independent factor, was negatively associated with participants' social connectedness scores (β = −0.13, *p* < 0.05). However, when the moderating effect of subjective attitudes toward esports gaming was considered, the association between communication frequency and social connectedness scores was turned into the opposite direction with a larger effect size (β = 0.35, *p* < 0.001).

**Conclusion:**

Our primary finding revealed that a positive mindset in esports gaming is indispensable in boosting social connectedness. Overall, our study provided supporting evidence for the benefits of esports on individuals' social connectedness. In future circumstances similar to the COVID-19 era, playing esports games is strongly encouraged in an attempt to maintain social connections and relieve psychological stress. In the meantime, we believe that having a positive esports experience, often associated with a positive mindset during gaming, can better promote social connectedness. Nevertheless, the amount of time spent on gaming per day should be of great concern, as esports games can be addictive, especially for teenagers and college students.

## 1. Introduction

### 1.1. Esports and its popularity

Unlike traditional sports in which physical maneuver is a primary component, esports is a unique type of sports that mainly depends on mental processing among participants (1, 2). It is carried out under unified competition rules in the virtual domain created by modern technology. Its gaming genres typically include Multiplayer Online Battle Arena (MOBA), Real Time Strategy (RTS), and First-Person Shooter (FPS), all of which rely on online multiplayer interactions (3, 4). Esports is immensely popular in many countries and often exceeds the popularity of traditional sports in viewership among young adults (5), who play a considerable role in esports market, especially in recent years (6). With the remarkable growth of esports globally, it has developed into a multi-billion-dollar business and continues to prosper (7). The global esports audience grew to 453.8 million worldwide in 2019 and is predicted to reach 645 million by 2022 (8). Economically, the total awarded prize money reached 150 and 211 million dollars in 2018 and 2019, respectively (9, 10). It has since attracted an enormous amount of qualified amateur players to pursue an esports career professionally (11, 12).

### 1.2. Esports and the COVID-19 pandemic

Esports has become a popular sporting mode as it could be held online without physical interaction among participants since the launch of social distancing and stay-at-home orders during the COVID-19 pandemic (13). During the COVID-19 lockdowns in 2020, online gaming participation significantly increased (14). For instance, the increased plays of Honor of Kings, one of China's most popular esports games, rendered a 20% increase in revenues in February 2020 (15). Some scholars proposed that the spike in participation in esports was a direct result of recurrent extended lockdowns and social distancing recommendations (14, 16–18). Moreover, live game streaming platforms were even used as an innovative medium to convey important COVID-19 health messages to people during the pandemic (19), indirectly revealing the popularity of video gaming, including esports, during the pandemic era.

### 1.3. Esports and social connectedness

Social connectedness, also referred to as social connection, can be thought of an individual's self-perception of closeness of interpersonal relationships with other people (20). Esports has become a practical avenue for people craving social connections during the COVID-19 public lockdown periods. The virtual world provides an alternative norm for socialization, so that people with social anxiety/phobia and social incompetence can compensate their social needs in a virtual way (21). Furthermore, esports games offered an accessible social space empowering participants for new friendships and maintaining or compensating for pre-existing social connections among young adults (22). Esports emphasizes a high level of interpersonal interactions because gamers oftentimes collaborate as a team for a common in-game challenge (23). This unique aspect of esports differs it from traditional video gaming by allowing a much more extent of socialization and interactions among participants (15, 24). Cole et al. found that massively multiplayer online role-playing games (MMORPGs) could foster social connections and enhance further social ties with more offline activities. Notably, some participants thus became life-long friends and partners (25).

The effects of esports participation on social interaction are equivocal. Unfriendly behaviors, such as online harassment and verbal abuse based on participants' gaming performance, virtual profiles and even races or ethnicities, negatively impact interpersonal social interactions (26). Märtens et al. noted the importance of preventing unfriendly communication behaviors by ensuring positive gaming experience and maintaining relationships between players (27). The negative effects of esports gaming on social connectedness have been observed when toxic communication occurs between players (28). In MOBA games, the participants from a losing team were more likely to feel ashamed and blame each other than those from the winning team (29, 30). Therefore, in this situation, social connections among esports participants could be worse rather than being consolidated. From the perspective of psychological wellbeing, if esports is not applied properly, it can be the subjects of addiction and hence have a negative impact on a person's mental health (31–35).

### 1.4. The present study

Generally speaking, these previous studies have shown that the pros and cons of esports participation on social connectedness are strongly modified by participants' behaviors. It was shown that subjective attitude has a powerful effect on individuals' behaviors during daily personal interactions (36, 37). However, it is unclear how it will affect such behaviors during esports participation. Furthermore, we suspect the potential impact of the COVID-19 pandemic on participants' behavior may further modify the relationship between esports participation and social connectedness.

Thus, the primary purpose of this study was to investigate how much subjective attitude toward esports affects the relationship between in-game interaction during esports participation and participants' anticipated social connectedness among Chinese young adults during the COVID-19 lockdown periods. Here we defined the in-game interaction by measuring the communication frequency during the course of esports participation. Consequently, we developed the following hypotheses, particularly in light of the COVID-19 lockdown periods:

- When controlling for relevant variables, increased communication frequency of esports participants can be negatively or positively associated with social connectedness.
- Subjective attitude toward esports gaming moderates the association between communication frequency and social connectedness.

## 2. Materials and methods

### 2.1. Overview

We conducted a questionnaire survey *via* the Credamo (38), a professional online survey platform by randomly inviting 18- to 25-year-old Chinese young adults to participate in this study. The survey period started on September 1, 2020, to September 30, 2020. The use of human data from the surveys was carried out ethically per the principles of the Declaration of Helsinki (as revised in 2013) (39). On the first page of the survey, all survey participants received an adequate description of the purpose and were asked to confirm an online informed consent before proceeding. All survey participants were explicitly asked in the consent to finish the survey to their best knowledge. All data was collected anonymously through the Credamo using serial identifier numbers to distinguish participants instead of recording their names or other sensitive information.

Two attention check questions at different time points in the survey were used for survey answering quality. A 10 RMB monetary incentive was offered for each valid completion. We manually checked the time taken to complete each survey and the responders' IP addresses to exclude replicate completions by the same participant. Only the following most popular esports games in China, which place a high emphasis on online multiplayer interaction, were included in the study: League of Legends (LOL), Defense of the Ancients 2 (DOTA2), Honor of Kings, Counter-Strike: Global Offensive (CS: GO), CrossFire (CF), Overwatch (OW), PlayerUnknown's Battlegrounds (PUBG), and PUBG Mobile.

### 2.2. Questionnaire contents

The questionnaire was comprised of the following information collected:

- Demographic information
- Esports platform (PC vs. Mobile), communication frequency during gaming and average daily gaming hours
- Subjective attitude toward esports gaming in influencing social connectedness (i.e., Do you think playing esports games can effectively upregulate interpersonal connectedness during the pandemic?)
- Social Connectedness Scale—Revised (SCS-R, 20 items)
- Whether participants experienced any non-esports gaming-related personal situations that had caused their social networking deteriorated severely.

### 2.3. Social connectedness assessment tool

The social connectedness of the participants was assessed using the 20-item version of the SCS-R. It measures experiences of intimacy in interpersonal contexts and difficulties in establishing and maintaining a sense of intimacy (40). Ten negatively worded items and the remaining 10 positively worded items are included on the scale. The negatively worded items are reversely scored, so a higher score of each item suggests a greater level of social connectedness and vice versa. The SCS-R can reach a total score comprised between 20 and 120 by using a six-point rating scale with 20 items. Participants were asked to choose the degree to which they agreed or disagreed with a statement on a scale of 1–6, on which 1 indicates “strongly disagree” and 6 indicates “strongly agree.” The Cronbach's alpha coefficient for this scale was 0.92, showing excellent internal consistency (41).

### 2.4. Statistical analysis

All statistical analyses were performed using the software program SPSS (version 26.0) except for the data cleaning process completed on the Credamo data platform. Reliability tests were conducted for the SCS-R, using Cronbach's alpha coefficients to measure internal consistency (α > 0.70 regarded as acceptable). Mean differences were compared by using parametric tests. The moderating effect of participants' subjective attitudes toward the relationship between esports participation and social connectedness was analyzed using hierarchical multiple regression. In the first step, gender, age, occupation, and exercise habit were entered into the regression equation to control the potential effects of other confounding variables. The second step included the variables related to esports gaming engagement during COVID-19. Finally, in the third step, the subjective attitudes of participants as a moderator variable on communication frequency were entered in the regression equation. Statistical significance is defined as a *p*-value < 0.05.

## 3. Results

### 3.1. Sample characteristics

Finally, a total of 550 young adults from mainland China enrolled in the survey. 52 were excluded for the failure in the attention check questions (e.g., responded wrongly to the instruction “please chose the answer Blue”); 25 were excluded for completing the survey in <200 s; 6 were excluded with additional analyses for other reasons such as answering the questionnaire questions inconsistently or contradictorily; 14 who experienced non-esports gaming-related personal situations that badly affected their social networking were also excluded to ensure following statistical analysis as reliable as possible. After stratification, a valid sample of 453 participants was analyzed collectively (261 females and 192 males; mean age = 21.51 years, SD = 1.93 years; age range: 18–25 years). The effective response rate was 82.4%. Relevant descriptive statistics were presented in Table 1.

**Table 1:** Sample description.

**Variables**	***n* (%)**
**Total**	453 (100.00)
**Demographic characteristics**	
Gender	
Male	192 (42.38)
Female	261 (57.62)
Age (mean 21.5 ± 1.9 years)	
Occupation	
Undergraduate	363 (80.13)
Post-graduate	46 (10.15)
Employee	44 (9.72)
Indoor exercise habit (over 20 min per day)	
Yes	171 (37.75)
No	282 (62.25)
**Esports games engagement characteristics**	
Gaming platform	
Based on personal computer	152 (33.55)
League of Legends	31 (6.84)
DOTA2	43 (9.49)
CS: GO	48 (10.60)
CrossFire	5 (1.10)
Overwatch	7 (1.55)
PUBG	18 (3.97)
Based on mobile phone	301 (66.45)
Honor of Kings	213 (47.02)
PUBG Mobile	88 (19.43)
Average time spent daily on esports games	
<1 h	55 (12.1)
1–3 h	215 (47.5)
3–5 h	137 (30.2)
5–7 h	29 (6.4)
Over 7 h	17 (3.8)
Communication frequency (through typing or audio)	
Never	20 (4.4)
Sometimes	187 (41.3)
Often	246 (54.3)
**Subjective attitudes on roles of playing esports games in**	
**affecting social connectedness**	
Has potential negative impact	4 (0.88)
Has no positive or negative impact	49 (10.82)
Be able to slightly improve	151 (33.33)
Be able to moderately improve	159 (35.1)
Be able to strongly improve	90 (19.87)

### 3.2. Mean comparison of social connectedness

Table 2 showed the social connectedness score comparison under different variables. Particularly, social connectedness scores were significantly different between three in-game communication frequencies (never, sometimes, and often) among all participants (*t* = 5.51, *p* < 0.005).

**Table 2:** Social connectedness (SC scores) of participants (*n* = 453).

**Variables**	**Social connectedness**			
	**Means (SD)**	**[95% CI]**	***t*/*F***	***p***
**Demographic characteristics**				
**Gender**			−1.51	0.133
Male	83.77 (13.79)	**[**81.81, 85.73**]**		
Female	81.87 (12.84)	**[**80.31, 83.44**]**		
**Occupation**			2.34	0.096
Undergraduate	82.37 (13.25)	**[**81.00, 83.74**]**		
Post-graduate	86.54 (11.92)	**[**83.00, 90.08**]**		
Employee	81.13 (14.25)	**[**76.80, 85.47**]**		
**Indoor exercise habit**			−3.40	0.001^**^
Yes	85.37 (12.78)	**[**83.44, 87.30**]**		
No	81.05 (13.32)	**[**79.48, 82.61**]**		
**Esports games engagement**				
**Gaming platform**			−1.01	0.312
PC	81.79 (13.74)	**[**79.59, 83.99**]**		
Mobile	83.13 (13.02)	**[**81.65, 84.60**]**		
**Daily gaming hours**			1.48	0.208
<1 h	81.67 (12.87)	**[**78.19, 85.15**]**		
1–3 h	82.02 (13.59)	**[**80.19, 83.85**]**		
3–5 h	84.44 (12.49)	**[**82.33, 86.56**]**		
5–7 h	79.37 (14.11)	**[**74.01, 84.74**]**		
Over 7 h	85.59 (14.17)	**[**78.30, 92.87**]**		
**Communication frequency**			5.51	0.004^*^
Never	82.60 (13.50)	[76.28, 88.91]		
Sometimes	80.28 (13.05)	[78.39, 82.16]		
Often	84.51 (13.18)	[82.85, 86.16]		

The total score on the Social Connectedness Scale—Revised ranges from 20 to 120.

^*^Indicates statistically significant at p < 0.05 level (2-tailed).

^**^Indicates statistically significant at p < 0.01 level (2-tailed).

### 3.3. Hierarchical multiple linear regression analysis

Table 3 showed all factors included in the regression analysis. Table 4 showed the hierarchical multiple linear regression model. No multicollinearity problem was detected in all three steps of the models. Our results showed that the mobile esports participants tended to have higher SC scores as compared to PC esports participants (β = 0.11, *p* < 0.005). Communication frequency as a separate factor was significantly yet negatively correlated with SC scores (β = −0.13, *p* < 0.05). When analyzing the moderating effect of subjective attitudes toward esports on the correlation between communication frequency and SC scores, it turned into a positive relationship with a larger effect size (β = 0.35, *p* < 0.001). All factors involved in the step 3 of the hierarchical model as a whole did not show a huge contribution in predicting the SCS-R scores (*R*^2^ = 0.10, Δ*R*^2^ = 0.05, Δ*F* = 25.01).

**Table 3:** Factors' values assigned in the hierarchical multiple linear regression analysis.

**Variables**	**Values**
Gender	1 = Female, 2 = Male
Age	Primary value
Occupation of participants	1 = Undergraduate, 2 = Postgraduate, 3 = Employee
Indoor exercise habit	1 = No, 2 = Yes
Gaming platform	1 = Based on personal computers, 2 = Based on mobile phones
Average daily gaming hours	1 = <1 h, 2 = 1–3 h, 3 = 3–5 h, 4 = 5–7 h, 5 = >7 h
Communication frequency	1 = Never, 2 = Sometimes, 3 = Often
Subjective attitudes^a^	1 = Negative impact, 2 = Neutral impact, 3 = Slightly positive impact, 4 = Moderately positive impact, 5 = Strongly positive impact
Social connectedness scores	Primary value

^a^Represents the subjective attitudes of participants toward playing esports games in influencing personal social connectedness.

**Table 4:** Hierarchical regression analyses predicting social connectedness (SC).

**Predictors**	***R* ^2^**	**Δ*R*^2^**	**Δ*F***	**Stand β**
**Step 1**	0.03^**^	0.03^**^	3.53^**^	
Gender				0.08
Age				0.01
Occupation				−0.04
IEH				0.16^***^
**Step 2**	0.05^***^	0.02^*^	3.44^*^	
Gender				0.11
Age				0.00
Occupation				−0.03
IEH				0.15^***^
DGH				0.00
GP				0.12^*^
CF				0.12^*^
**Step 3**	0.10^***^	0.05^**^	25.01^***^	
Gender				0.10
Age				−0.02
Occupation				−0.03
IEH				0.16^***^
DGH				−0.02
GP				0.11^**^
CF				−0.13^*^
SA × CF^a^				0.35^***^

CF, communication frequency; DGH, daily gaming hours; GP, gaming platform; IEH, indoor exercise habit; SA, subjective attitudes.

R^2^ = 0.03 for Step 1; ΔR^2^ = 0.02 for Step 2; ΔR^2^ = 0.05 for Step 3.

^a^SA represents subjective attitudes of participants toward playing esports games in influencing personal social connectedness.

Betas were standardized. ^*^P < 0.05; ^**^P < 0.01; ^***^P < 0.001.

## 4. Discussion

Previous studies showed that a higher socially interactive environment could be achieved by virtual gaming because the virtual domains could provide a more comfortable avenue for interpersonal interaction without the disclosure of personal identities (15, 22, 42). This may provide a positive benefit for communication in improving social connectedness in esports. However, our results initially suggested that participants with a higher social connectedness level were less likely to have higher communication frequency, when controlling other variables (i.e., gender, age, occupation, indoor exercise habit, gaming platform, and average daily gaming time). Nevertheless, after considering the subjective attitude as a moderating factor, this negative correlation turned into a stronger yet positive relationship. This indicated that possessing positive attitudes toward esports gaming played a significant role in promoting personal social connectedness. A reasonable explanation for these findings could be that interaction among esports participants does not necessarily mean that communication is always in a positive manner. This aligned with the above-mentioned cons of esports on social connectedness (26–30). Moreover, the emotional burdens during esports, such as the stress from losing a game or failure to cooperate, could further exacerbate the adverse outcomes that the in-game communication yields (28). Therefore, the introduction of a subjective attitude toward esports is important in ruling out the potential underlying cause of the negative correlation between in-game communication frequency and social connectedness. Our results showed that esports participants with a positive attitude and higher communication frequency tended to have a higher social connectedness level during the COVID-19 pandemic.

The subjective beliefs of participants are crucial in improving social connectedness. This causal effect may be partially explained by the close interactions between attitudes and behaviors. Attitudes can be powerfully and imperceptibly influential over behaviors (36, 37). Because of cognitive dissonance, people are likely to alter their behaviors to align them with their attitudes better when experiencing conflicting attitudes and behaviors (36). Also, people's feelings as feedback resulting from their desirable behaviors can make their pre-existing beliefs either strengthen or shift in the opposite direction (37). Yet the latter condition is less likely to occur in the context of esports gaming, which is very individually mindset-oriented, regardless of the result of winning or losing a game (43). Hence, for instance, having subjectively positive attitudes toward esports participation can motivate gamers to combat and cooperate with others in a more positive and friendly manner during communication through audio or chatting. These behaviors can be further reinforced with increased frequency of communication, therefore jointly creating a higher level of personal social connectedness.

On the other hand, negative attitudes can induce an adverse outcome regardless of the communication frequency. Thus, far, our study bridged the gap in how esports gaming impacts personal social connectedness, especially during the COVID-19 pandemic. Taken together, possessing positive mindsets toward esports gaming is an overriding priority over other factors in promoting personal social connectedness.

Nonetheless, although it appears from our findings that more positive in-game interactions theoretically lead to better social connectedness, regardless of other factors (even with excessive daily gaming hours), we believe that across all types of games, more than 3–5 h of gaming time per day can be detrimental to personal mental health (44), and esports games are no exception. For instance, Sunil et al. suggested that playing PUBG may affect the psychological development of young people, and somehow contribute to their self-harm and suicidal behaviors, which are attributed to the violent nature of PUBG (45). In addition, a long-term sedentary lifestyle is not conducive to physical health (46). Thus, daily gaming time should be of high concern, especially for adolescents and college students.

The hierarchical model in Table 4 did not show an obvious contribution in predicting the SCS-R scores of participants (*R*^2^ = 0.10), but this could be understandable because playing esports games alone could not determine the overall social connectedness of individuals, and this study was not trying to achieve that, but a correlational study of potential factors that may affect a person's social connectedness.

Overall, to the best of our knowledge, this is among the first studies to explore the effects of esports participation on social connectedness based on in-game communication frequency and exclusively taking participants' subjective attitudes into consideration. We particularly analyzed participants' psychological parameters during the COVID-19 pandemic. Our study may yield a different conclusion than older ones. Table 5 listed our recommendations for some practical suggestions related to our studies. We believe that a positive esports experience can yield better social connectedness and, therefore, more positive psychological wellbeing (48, 49).

**Table 5:** Practical recommendations of the present study.

**Main suggestions**
• In the future, when staying indoors is encouraged or mandated in response to a particular situation, playing esports games as a safe and convenient entertainment is highly recommended to cope with stress (47).
• For people without previous gaming experience, it is preferable to try mobile gaming first. It does not take as much as the time compared to PC gaming, so the amount of the hours spent on games is easier to control.
• We strongly encourage avoiding negative communication behaviors and showing positive attitudes (e.g., praise and encouragement) when interacting with other players during esports gaming.

We used communication frequency to reflect participants' in-game interactions. Together with subjective attitudes, they may indirectly reflect the emotional tones of participants in communication during esports participation. If applicable, we encourage future research to directly investigate the role of emotional tone of communication behavior during gaming in influencing social connectedness and psychological wellbeing among players.

## 5. Limitations

The current study has several limitations:

- The questionnaire survey was not delivered immediately following China's pandemic lockdown periods. The time delay may influence the accuracy of results because the social networking of participants could change due to potential personal or social factors due to the dynamic characteristics of the pandemic.
- The study was designed to survey participants during a unique period of time. Our results may not be applicable under general situations or replicated in the future.
- This study was based on self-reported responses of young adults.

## 6. Conclusion

The demand for participation in esports gaming has been prospering. More young people are attracted to esports as alternative entertainment during the pandemic. Under certain conditions, esports participation can be a valuable way of fostering social connectedness when in-person activity is not permitted. However, their subjective attitudes strongly affect the relationship between esports participation and personal social connectedness. Based on the present study, we conclude that increasing participation through more frequent and positive communication with other players when playing esports games is beneficial for building social connectedness.

## Data availability statement

The original contributions presented in the study are included in the article/supplementary material, further inquiries can be directed to the corresponding author.

## Ethics statement

Ethical review and approval was not required for the study of human participants in accordance with the local legislation and institutional requirements. The participants provided their written informed consent to participate in this study.

## Author contributions

DS was primarily responsible for the overall content of the article, including independently drafting the initial version of manuscript. JX hugely contributed to all aspects of the current article during multiple revision phases. TL greatly contributed to data analysis and interpretation of the results. YZha and ZiD contributed to data collection and minor revisions of the manuscript. YZhe and YW participated in the literature search and manuscript editing. CL and ZhD made some critical revisions to the manuscript. All authors have read and approved the final version of the manuscript, agree with the order of the presentation of the authors, and contributed significantly to this article.
